# Preparation of Chalk for Dental Powders

**Published:** 1888-11

**Authors:** 


Preparation of Chalk for Dental Powders.—Dissolve 1 part of chloride of calcium (muriated chalk) in 15 parts of distilled water; filter it and add a filtered solution of crystallized soda and distilled water, until it will not produce any more deposit. The white deposit which forms at the bottom by that time is the precipitated chalk. After the solution has stood for a time, decant, then the wet deposit is placed in a funnel on filter paper, and syringed six or eight times; then left to dry. The powder thus obtained is entirely free of sand, and an excellent dentifrice may be made as follows:

| | | |
|---|---|---|
| R. | Precipitated chalk - - - - - | 250 grains. |
| | Pulv. violet root - - - - - - | 125 " |
| | Carmine - - - - - - | 4 " |
| | Essence of rose - - - - - - - | ½ " |
| | Essence of sandal - - - - - | 10 drops. |

*From Neueste Erfindungen.*

